# How I Would have been Differently Treated. Discrimination Through the Lens of Counterfactual Fairness

**DOI:** 10.1007/s11158-023-09586-3

**Published:** 2023-03-20

**Authors:** Michele Loi, Francesco Nappo, Eleonora Viganò

**Affiliations:** 1 Department of Mathematics, Politecnico Di Milano, Milan, Italy; 2 Institute of Biomedical Ethics and History of Medicine (IBME), University of Zurich, Zurich, Switzerland

**Keywords:** Theories of discrimination, Counterfactuals, Counterfactual fairness

## Abstract

The widespread use of algorithms for prediction-based decisions urges us to consider the question of what it means for a given act or practice to be discriminatory. Building upon work by Kusner and colleagues in the field of machine learning, we propose a counterfactual condition as a necessary requirement on discrimination. To demonstrate the philosophical relevance of the proposed condition, we consider two prominent accounts of discrimination in the recent literature, by Lippert-Rasmussen and Hellman respectively, that do not logically imply our condition and show that they face important objections. Specifically, Lippert-Rasmussen’s definition proves to be over-inclusive, as it classifies some acts or practices as discriminatory when they are not, whereas Hellman’s account turns out to lack explanatory power precisely insofar as it does not countenance a counterfactual condition on discrimination. By defending the necessity of our counterfactual condition, we set the conceptual limits for justified claims about the occurrence of discriminatory acts or practices in society, with immediate applications to the ethics of algorithmic decision-making.

## Introduction

Much philosophical literature on discrimination has traditionally focused on what makes a discriminatory act or practice wrong, rather than on the prior question of what makes an act or practice an instance of discrimination (see, e.g., Alexander 1992, p. 159; Lippert-Rasmussen 2014, ch. 6; Hellman 2008, ch. 1; 2018, pp. 100–104; Eidelson 2015, chs 3–4). After all, most of us have an intuitive idea about what discrimination is and can point to cases of discrimination, such as apartheid in South Africa. What has mostly attracted the interest of philosophers, then, are the properties that make discrimination morally wrong. However, as recent historical developments show, in many real-world situations the question of what constitutes discrimination is by far the most important one. This issue is becoming particularly pressing with the diffusion of prediction-based decision algorithms, viz., algorithms that utilize the tools of machine learning to help human decision-makers to make decisions in fields such as employment, crime prediction, and loan assessment. When designing such algorithms, it is of the utmost importance to avoid the introduction or perpetuation of discriminatory practices.

In the machine learning literature, Kusner et al. (2017) have developed a formal approach for assessing prediction-based decision algorithms, known as counterfactual fairness. Adopting the influential causal modeling framework developed by Pearl (2000, 2009), Kusner et al. (2017) propose to consider how membership of a given demographic group affects the distribution of benefits and costs of a given policy or decision. While their account has received criticism from both computer scientists and philosophers (cf. Chiappa 2019; Castro 2022; Castro et al. 2022), our aim in this contribution is to provide a defense of one important aspect of it, which we believe has not received the philosophical attention that it deserves. This is the idea that discrimination implies that a counterfactual condition is satisfied: in short, for an individual to have a claim to be discriminated against by a given procedure or practice, it must be the case that, had that individual belonged to a different socially salient group (in terms of, e.g., gender or race), the probability of receiving the same treatment that they received would not have been the same. Our main claim is that this condition is conceptually necessary, although not sufficient, for an act or practice to be an instance of discrimination. We will refer to acts and practices that satisfy the counterfactual condition as instances of K-discrimination.

The notion of K-discrimination constitutes, in our view, the conceptual core of acts and processes of morally wrongful discrimination and is, for this reason, philosophically relevant. We argue for this thesis by showing that two of the most prominent accounts of wrongful discrimination in the recent philosophical literature, by Lippert-Rasmussen (2014) and Hellman (2008, 2018) respectively, face objections precisely insofar as they deviate from K-discrimination. Specifically, Lippert-Rasmussen’s account ends up treating as discriminatory acts or practices that should not be understood as instances of discrimination at all, as when the allegedly discriminated individual does not actually belong to the socially salient group that a practice discriminates against. Hellman’s account, instead, turns out to lack explanatory power in diagnosing instances of discrimination insofar as it does not (at least officially) include a counterfactual condition. By defending K-discrimination as a prerequisite for discrimination, we aim to set conceptual limits for justified claims about the occurrence of discriminatory acts and practices in society—including, but not limited to, those involving prediction-based algorithms.

Our discussion also provides a novel key to the debate over the use of fairness measures in machine learning. Reviewing the failures of various prominent proposals to operationalize fairness in algorithmic predictions, Castro (2022) and Castro et al. (2022) have contended that that project is likely impossible and even wrong-headed. Although our thesis is not intended as a refutation of their view, we defend the fruitfulness of the counterfactual approach by Kusner and colleagues to the debate on what constitutes discrimination. To show this, we introduce some relevant conceptual distinctions in the discrimination vocabulary, identify the types of discrimination that K-discrimination captures, and discuss its relationship with counterfactual fairness. If we are right, the type of empirical test for fairness proposed by Kusner et al. (2017) has indeed a tight conceptual relationship with one morally relevant form of unfairness, namely discrimination. This does not make counterfactual fairness the only or ultimate criterion of fairness, since [as Castro et al. (2022) note] the latter is spoken of in many ways.

The plan for the paper is as follows. In ‘The Counterfactual Approach’ section, we will present the counterfactual fairness approach by Kusner et al. (2017) and isolate the aspect of it that we regard as the philosophically most interesting and consequential: the idea that discrimination (suitably qualified) is subject to a counterfactual condition. In ‘Clarifying K-Discrimination’ section, we will clarify K-discrimination by proposing conceptual distinctions within the discrimination realm, also providing further details regarding the relevant counterfactual comparisons. In ‘The Necessity of K-Discrimination, Part I’ section, we will show how K-discrimination escapes the various objections that have been posed to Kusner et al.’s (2017) approach, both with regards to its formulation and its verdicts about relevant case-studies. In ‘The Necessity of K-Discrimination, Part II’ section, we will argue that philosophical theories of discrimination that do not imply the necessity of K-discrimination are subject to important objections, using Lippert-Rasmussen’s and Hellman’s accounts as examples. In ‘The Conceptual Limits of Discrimination’ section, we will discuss further issues for our account which stem from its association with a broadly interventionist view of causation and will illustrate how our account adheres with sensible norms regarding the attribution of responsibility in practice. In ‘Conclusion’ section, we will conclude with a summary of our arguments.

## The Counterfactual Approach

In response to a growing need for a credible measure of fairness for machine learning predictors, Kusner et al. (2017) have proposed a formal approach based on counterfactuals. Their account aims to capture the idea of fairness as sameness of treatment across demographic groups; or, more specifically:

The intuition that a decision is fair towards an individual if it is the same in (a) the actual world and (b) a counterfactual world where the individual belonged to a different demographic group. (Kusner et al. 2017, p. 1)

This approach promises to apply to predictions or decisions of so-called ‘blind’ algorithms, i.e., rules that are applied based on data that do not include explicit information about group membership (Kilbertus et al. 2018; Žliobaitė and Custers 2016). Algorithms of this kind are viewed as incorporating a model of the way in which being member of a demographic group shapes the probabilities of a person’s acquiring features that are relevant to the algorithm’s predictions. According to Kusner et al. (2017), counterfactually fair algorithms can compensate for the biases that are based on socially sensitive attributes and that could bring about discrimination toward individuals or groups.

The rationale behind Kusner et al.’s (2017) proposal is the following. Many proposed fairness measures in machine learning fail to distinguish between correlation and causation. As discussed by Glymour and Herington (2019), both outcome-relative (e.g., separation, equality of false-positive rates, equality of false negative rates) and score-relative (e.g., sufficiency, positive predictive value parity, negative predictive value parity) definitions of fairness imply unfairness when the correlation between group membership and treatment is not causal. In other words, those rival criteria do not differentiate when group membership and prediction are causally connected directly, indirectly, or by way of a common cause. Yet, clearly, a mere correlation between group membership and a favorable prediction does not necessarily indicate a causal influence of the former on the latter. For instance, the fact that more men than women are admitted at a given school need not illustrate an effect of gender on the selection process, if we later find out that significantly many more men than women applied. Requiring a criterion of counterfactual dependence on fairness implies, according to Kusner et al. (2017), that one asks whether, keeping other factors fixed, an intervention on the group membership variable would have made a given treatment prediction more or less probable for an individual.

To formalize these ideas, Kusner et al. (2017) begin by interpreting membership to a demographic group as taking a specific value within a set of observable protected attributes (denoted by A). The attributes over which A ranges are primitives, i.e., they are established a priori. In the style of Pearl (2000), one then builds up a causal model relative to the problem situation, which represents the events that occur in that situation together with the relations of counterfactual dependence between them. A problem situation is thus analyzed in terms of three kinds of variables. First, we have the class of variables representing the features of an individual that are not causally influenced by group membership. Then, we have the intervention variable: the feature one must intervene upon in order to test a hypothesis about discrimination, e.g., membership of a given gender. Finally, we have the variables that are affected causally by the intervention variable. This includes all those features of the individual that are relevant to their treatment by a given act or policy (e.g., what type of role models one gets exposed to, the probabilities of selecting a course at the university as opposed to another one) and which are expected to vary as a result of a change in the intervention variable in accordance with the relations of counterfactual dependence in place between them. Relative to the causal model thus obtained, a predictor—i.e., the (human or artificial) entity making a prediction about an individual—is said to be ‘fair’ if the probability of this individual receiving a certain treatment is not altered by manipulations on the intervention variables.

In the philosophical literature, Kusner et al.’s (2017) approach has been met with skepticism. The main problems stem from its being originally intended as a *theory* of fairness. First, Kusner et al. (2017) refer primarily to predictors rather than decisions; accordingly, their account is bound to treat certain algorithms as discriminatory even when they do not bring about any outcome (Fleisher 2021). Secondly, the theory’s purely formal understanding of membership to a demographic group as taking a value within a set of protected attributes seems to allow for significant gerrymandering of the morally relevant groups (see also ‘Lippert-Rasmussen’s Group Discrimination with a Real or Perceived Trait’ section). Finally, the implication that counterfactual fairness is necessary for fairness is subject to obvious counterexamples. As first noted by Chiappa (2019), there are legitimate paths of causal influence from membership of a demographic group that should not count toward a policy’s unfairness. Castro (ms.) illustrates this point by the example of a company looking for an employee that is fluent in Spanish and where the relevant qualification is assessed through a language test. If we imagine a candidate, Simon, who is non-Hispanic and did not study Spanish, it is plausible that he will not pass the language test. However, in the counterfactual world in which Simon belongs to the demographic group of Hispanics, he would pass the test. By Kusner et al.’s (2017) account, the language test is an unfair predictor. Intuitively, however, the company’s adoption of the language test is not unfair since Simon is not treated differently insofar as he is non-Hispanic.

While we agree with the criticisms that counterfactual fairness errs in the above respects, we do not think that this is the end of the story. As anticipated, our contention is that the theoretical framework of counterfactual fairness is most usefully seen as a contribution to the problem of what constitutes discrimination (as a specific form of unfairness). In particular, we contend that an important necessary condition on discrimination can be extracted from the above approach, as follows:

A causal process results in an instance of discrimination toward an individual only if it does not lead to the same treatment with the same probability in (a) the actual world and (b) in a nearby counterfactual world where the individual belongs to a different socially salient group.

We define ‘K-discrimination’ as the property of an outcome of any causal process that does not lead to the same treatment with the same probability for an individual in (a) the actual world and (b) in a nearby counterfactual world where (in a sense to be specified further in the next section) the individual belongs to a different socially salient group. Our thesis will be, accordingly, that the outcome of a causal process can be an instance of morally wrongful discrimination only if it is an instance of K-discrimination.^1^

By claiming that K-discrimination is a necessary condition on discrimination, we claim to isolate the most important insight behind the counterfactual fairness approach. Indeed, as the next sections will illustrate, the thesis that discrimination implies K-discrimination is not only immune to the counterexamples often raised against the counterfactual fairness approach, but is also highly relevant to the moral problems raised by the adoption of machine learning prediction algorithms. There is, however, an accompanying intuition in Kusner et al.’s (2017) approach that we aim to highlight here, and which we retain in our proposal. This is the idea that what brings about discrimination, in the first instance, is not a single decision, but rather the entire causal process that generates some decisions. Hence, for us, individual acts that result from a causal process that brings about discriminatory outcomes are only discriminatory in a derivative sense; social practices fall within the scope of K-discrimination insofar as they can be modeled as causal processes. The salient property that K-discrimination isolates as a prerequisite for discrimination is therefore a causal process’s generating outcomes biased towards some socially salient groups. This condition is obviously extendable to decision processes other than intentional ones. Thus, a prediction, as it happens in the case of algorithmic decision-making, or a decision due to an unconscious attitude, such as in cases of implicit bias, can be outcomes of causal processes and, as such, satisfy K-discrimination.

We will elaborate on K-discrimination’s definition and its role as a prerequisite for a morally relevant notion of discrimination in ‘Clarifying K-Discrimination’ section. Before that, let us rely on the reader’s informal grasp of the proposed condition to illustrate its work in a pair of examples. Consider the following:

*Machinery*—A company introduces new machinery that requires workers to be at least 1.75 m tall to operate. As a result, women are disproportionately affected by the company’s decision compared to men: in fact, many of them are forced to leave their job.

*Aetastopia*—In the imaginary world of Aetastopia, age is the only factor influencing the probability that a person released on parole will reoffend. However, age distribution for men in Aetastopia differs from women’s. As a result, an age-sensitive algorithm for awarding parole decisions leads to an unequal rate of favorable decisions for men compared to women.

Arguably, there is a form of discrimination against women in machinery (although, as we will discuss in ‘Clarifying K-Discrimination’ section, a further question may be raised as to whether this discrimination is all-things-considered morally wrong); conversely, no such discrimination occurs in Aetastopia. The difference is captured by K-discrimination: in machinery’s case, if we keep the actual causal structure of the world (including the known biological influences of sex on height) and just imagine a counterfactual world in which an individual’s sex is switched from female to male, the probability of that individual keeping their job at the company is raised significantly. Conversely, the decision procedure in Aetastopia is not discrimination (but mere disparate impact). This is because, by hypothesis, an individual’s being a woman is not causally related to either age or probability of reoffending. Hence, if we imagine switching the individual’s ‘gender variable’ to woman while keeping the causal structure fixed, the probability of favorable parole decision remains the same.

Note that by denying that Aetastopia is a case of discrimination, we do not imply that its parole decision system is necessarily ‘fair’. For instance, an unequal rate of false positives in the reoffending predictions between men and women may be regarded as a form of inequality of opportunity and hence of ‘unfairness’ for that reason (cf. Heidari et al. 2019; Castro et al. 2022). The strength of K-discrimination lies with its being a much narrower thesis, which is compatible with a great variety of philosophical positions regarding the notion of fairness and its operationalization in machine learning. This observation will be taken up in section ‘The Necessity of K-Discrimination, Part I’, where we will address the criticisms of Castro (2022) and Castro et al. (2022) of counterfactual fairness. Before that, some clarifications are needed concerning how the relevant counterfactual comparison must be performed in order to assess whether K-discrimination is satisfied. Addressing this important set of philosophical questions is the main task of the next section.

## Clarifying K-Discrimination

The concept of discrimination can be used in a moralized and a non-moralized way: in the former case, one means discrimination that is necessarily morally wrong; in the latter, one means a certain type of action that is morally neutral. There is an obvious distinction in terms of moral relevance, for instance, between the expressions ‘a discriminating law’ and ‘a discriminating taste for wine’. As a matter of fact, this initial distinction is not sufficient to capture the finer structure of conceptual relations between discrimination and moral wrongness. One further distinction we should draw with regard to the moralized use of discrimination is between pro tanto and all-things-considered morally wrong discrimination. To illustrate, machinery is (by our lights, at least) a case of pro tanto morally wrong discrimination, meaning that the company’s decision is wrong in some respects in virtue of, and precisely to the extent that, it is discriminatory. However, by filling in more details about the company’s decision, we may come to the conclusion that their decision, albeit discriminatory, is not morally wrong all-things-considered. For instance, if the machinery’s being introduced is required by new safety regulations (and no other machinery is available), we may come to regard the reasons in favor of its adoption to override the reasons against it (viz., its discriminating against women).

A second distinction to draw is between prima facie and ultima facie discrimination. This is the distinction between something that merely appears to be discriminatory (and can be wrong for that reason) and an act or practice that is indeed discriminatory (and can be wrong for that reason). For instance, the unequal rate of favorable parole decisions in Aetastopia may be regarded as prima facie discriminatory even though further reflection shows that it is not discriminatory—and hence it is not an instance of ultima facie discrimination. (Of course, as we discussed, Aetastopia’s parole decision system may be unfair for other reasons, and so ultima facie wrongful for that reason, but not in that it is discriminatory.) This contrasts with machinery, in which we do not have just the appearance of a disparate impact along a protected attribute (and hence a case of prima facie discrimination), but also a case in which we realize that a process is indeed discriminatory (it is thus a case of ultima facie discrimination). It is a consequence of the above-mentioned distinctions that if an act or practice is discriminatory (either pro tanto or all-things-considered), then it is necessarily an instance of ultima facie discrimination. For all we said, however, there may also be acts or practices that count as ultima facie discrimination but fail to be morally wrong (even pro tanto). As we will clarify in ‘Lippert-Rasmussen’s Group Discrimination with a Real or Perceived Trait’ section, our distinctions regarding prima and ultima facie discrimination are only superficially related to those introduced by Lippert-Rasmussen (2014). We will consider these differences in approach when they become relevant to our argument.

The specific claim that we aim to defend in this contribution is that K-discrimination constitutes a necessary condition for ultima facie discrimination that is at least pro tanto morally wrongful. As such, K-discrimination helps shed light on an important connection between the concept of discrimination and the moral status of discriminatory practices—one that philosophical discussions on the wrong-making features of discrimination have often failed to bring out. This is the point that when morally wrongful discrimination occurs (either pro tanto or all-things-considered), K-discrimination’s being satisfied is arguably part of the explanation for why that act or practice is morally wrong. For instance, it is part of the explanation for the wrongfulness of the company’s decision in Machinery that, had there been men in place of the affected women, they would not have had the same chance of losing their job due to height limitations. Different theories of the wrong-making features of discrimination may, of course, complete this explanation in different ways. For instance, harm-based accounts would add that the discriminatory act makes people worse-off (e.g., Lippert-Rasmussen (2014)); respect-based accounts (e.g., Hellman (2008)) would point to the fact that the company’s act is demeaning. On our view, however, no theory has potential to explain the special moral wrongness of discrimination in isolation from the necessary condition that we identify.

As K-discrimination makes essential use of technical vocabulary of counterfactuals, let us proceed to clarifying these aspects of the proposed condition immediately. First, K-discrimination’s appeal to ‘socially salient groups’ should be clarified. In this regard, we borrow Lippert-Rasmussen’s (2014) definition of a socially salient group as a group such that ‘perceived membership of it is important to the structure of social interactions across a wide range of social contexts' (p. 30). The idea is that in the actual world membership of some groups robustly shapes the nature and quality of one’s social interactions. As to which exact groups are socially salient in the relevant sense, we entirely defer to a background sociological theory. It is a task for such a theory to tell us which groups are socially salient at a given time in a given society and how best to individuate them—if, for instance, the category of ‘black woman’ should be treated as a separate socially salient group in a society at a given time, or as merely a case of double membership to two socially salient groups, viz., ‘blacks’ and ‘women’. Of course, the relevant sociological facts are liable to change across time and contexts. One might plausibly argue, for instance, that the group of ‘Aryans’ was a socially salient group in Nazi Germany as the result of the way in which membership of that group was defined in social and legal terms and shaped an individual’s rights and opportunities within that society.

A ‘nearby counterfactual world’ must then be intended as a world in which the basic meanings and sociological facts about each socially salient group remains the same as the actual world, but where some facts about individual memberships of groups change. In terms of the causal modeling framework, a nearby world is one in which the background variables and relations of counterfactual dependence are the same as the actual world—so as to leave the causal structure fixed. K-discrimination then requires us to evaluate what would have happened to an individual if, keeping this causal structure fixed, they had belonged to a different socially salient group. Such a world is ‘counterfactual’ solely with regards to the group membership of the individual and everything that follows causally from that fact. Accordingly, K-discrimination excludes from the relevant comparison ‘far away’ counterfactual worlds, including (but not limited to) those in which the causal role of the group, by virtue of which it qualifies as socially salient, is different from the actual one. In other words, K-discrimination does not ask what would have happened to an individual had there been in place a society different from ours in some basic respects (such as in its attribution of social meanings to perceived membership of socially salient groups), but only what would have happened in a society relevantly like the actual one in question except for some slight change in the features that some individuals happen to possess. As we will elaborate in ‘The Conceptual Limits of Discrimination’ section, the justification for this restriction must ultimately be found in the conceptual limits of what we can meaningfully refer to as ‘discrimination’.

Within the family of the nearby worlds, we do not need to specify what the most relevant contrast or the ‘closest’ counterfactual world would be in any given case. In fact, K-discrimination requires that we consider all the nearby worlds in which the group membership feature takes a different value. If an individual does not receive the same treatment with the same probability in at least one such nearby world, then we have K-discrimination. For instance, in evaluating whether a Jewish professor was discriminated by being denied an academic position in Nazi Germany, we ought to ask whether he or she would have received different treatment in at least one nearby world. That amounts to asking whether belonging to any other socially salient group (in every nearby world) would have led to the same treatment with the same probability. Of course, in some nearby possible worlds the individual belongs to another discriminated group (e.g., Roma). However, it is sufficient for K-discrimination to be satisfied that there is at least one world in which, with a different group membership, the probability of hiring the professor would have been different. Furthermore, it is sufficient that this probability be different, not that it is necessarily greater or smaller. Insisting on a more specific claim is unnecessary for our purposes because, as we emphasized earlier, we are not offering K-discrimination as a sufficient condition for pro tanto wrongful discrimination, but only as a necessary condition. The point that concerns us the most, and that we find to be philosophically highly significant, has to do with the restriction of the worlds being considered to the ‘nearby’ possible worlds. It is differential treatment relative to that specific strip of the logical space of possibilities that we regard as necessary for pro tanto wrongful discrimination.

It may still be objected that socially salient features such as race are in some sense ‘essential’ to an individual and that, therefore, no sense can be made of at least some of the counterfactual comparisons that K-discrimination requires us to make (cf. Kripke 1980). However, the philosophical questions about the identity of individuals in counterfactual worlds can be side-stepped as inessential to our argument. In order to determine whether K-discrimination is satisfied, we do not have to identify a counterpart of the actual individual in every nearby possible world. It is sufficient that we can evaluate the treatment and its probability for all individuals who share the same features as the actual individual except for the group membership and all features that causally downstream from it. For instance, we do not ask what the probability of being hired is for Frank, who is actually Jewish, in a world in which he is not Jewish. Rather, we ask what the probability is for those who share with Frank certain features but not others. This is plausible because what concerns us the most in cases of discrimination is not so much whether an individual identical to the one in question, but for the group membership trait, would have been treated differently; rather, it is whether an individual sharing relevantly similar characteristics (e.g., similar competences for a position) would have received a different treatment merely as the result of a difference in membership of a socially salient group.

To summarize, in this section we have clarified our thesis that K-discrimination is a necessary condition on morally wrongful discrimination. As we hope to have illustrated through this discussion, a defense of K-discrimination is highly relevant for the debate on the nature and moral status of discrimination. This is because it identifies certain types of processes as potentially morally wrongful and, as such, constitutes the basis upon which a moral theory of discrimination can be built. In the next section, we are going to defend our thesis from a number of existing objections in the literature.

## The Necessity of K-Discrimination, Part I

Recent discussions by Castro (2022) and Castro and colleagues (2022) provide a recipe for generating counterexamples to any measure or test for fairness as applied to prediction-based algorithms, including the counterfactual fairness approach. The recipe consists in finding a context in which the alleged conditions for fairness or unfairness are satisfied, but the intuitive verdict diverges. In particular, Castro (2022) and Castro et al. (2022) provide objections both to the claim that counterfactual fairness is sufficient for fairness and to the claim that it is necessary. This urges us to consider whether the same objections extend to our thesis. After all, it may be supposed that the notions of unfairness and discrimination are intimately connected. As a result, one might suppose that the proposed counterexamples to counterfactual fairness extend to our account. However, in what follows we will demonstrate that these expectations are incorrect. We will provide reasons to think that, as a matter of fact, pro tanto morally wrongful discrimination is the kind of concept for which it is plausible that an operationalization exists—or, at least, that the notion is subject to the conceptual constraints of which K-discrimination is precisely the expression.

Let us start by considering the case of the Spanish test discussed in ‘The Counterfactual Approach’ section. As Castro (ms.) notes, this is a counterexample to the idea that fairness implies counterfactual fairness. Intuitively, there is no unfairness toward Simon (who is not a Spanish speaker) in requiring a Spanish entrance test despite the latter’s failing the condition of counterfactual fairness. This refutes the attempt to define fairness in terms of counterfactual fairness. To see why this is not a problem for our account, however, let us concede the following bridge claims for the sake of the argument:

(A) If there is fairness (F), then there is no discrimination (not-D).
(B) If there is no counterfactual fairness (not-KF) then there is K-discrimination (KD).

We can then immediately reformulate Castro’s argument as objecting to the thesis that the absence of discriminatory treatment (not-D) implies the absence of K-discrimination (not-KD) or, contrapositively, that K-discrimination (KD) implies discrimination (D). This is patently not our thesis: as already stressed, the direction of our thesis is the opposite, viz., (D) implies (KD).^2^

One might still expect that Castro-style objections to the opposite direction of implication would provide a counterexample to our view. Consider, for instance, Castro et al.’s (2022) example of Law School Success, in which, seeking a fair criterion for law school admissions, a university’s admission team uses passing a pre-law course as a determining factor for admission; however, it just so happens that only one of the two colleges from which the university’s applicants come provides the opportunity to attend the pre-law course. Castro et al. (2022, p. 18) add that they are ‘perfectly integrated’, by which we understand that the probability of attending either college is not affected by membership of any socially salient group. This plausibly suggests that counterfactual fairness (KF) does not imply fairness (F). However, to turn this into a problem for our thesis, two further assumptions are needed:

- (A*) If there is no fairness (not-F), then there is discrimination (D).
- (B*) If there is counterfactual fairness (KF), then there is no K-discrimination (not-KD).

In general, we reject A*. Law School Success illustrates this well: although the case may be regarded as a blatant violation of equality of opportunity by some, and therefore ‘unfair’ for that reason, there is scarcely any evidence of discrimination properly speaking. After all, by hypothesis the colleges are ‘perfectly integrated’ (Castro et al. 2022, p. 18). As already discussed in the case of Aetastopia, fairness (F) and absence of discrimination (not-D) may come apart in interesting ways.

Castro (2022) offers a different type of example to counterfactual fairness being sufficient for fairness. This is his SUNDIAL case: a system that decides whether to release people on parole that ‘bases its prediction of recidivism off of two features of defendants that are not casually influenced by any protected attributes: law knowledge and mischievousness' (Castro 2022, p. 176). SUNDIAL labels as high-risk, people who possess both features. As the result of the police’s choice to monitor certain schools more often than others, there are no white people with law knowledge that are screened by SUNDIAL and many black people with law knowledge that are. Consequently, the system labels as high-risk, innocent black people more often than innocent white people. Once again, this suggests that counterfactual fairness is not sufficient for fairness, since SUNDIAL is by hypothesis counterfactually fair but a form of unfairness results from its use. Moreover, in this case it seems highly plausible to describe the use of SUNDIAL as discriminatory. If this is correct, then we have a counterexample to the necessity of K-discrimination.

However, we believe that SUNDIAL does not constitute a problem for our view. In fact, the case is under-described. At least two versions of the case may be distinguished. In the former, SUNDIAL*, the positive correlation between law knowledge and being black has a common cause, namely the racist bias in policing practices (whereby they enter predominantly black schools but never access predominantly white ones). In the latter, SUNDIAL**, it is simply an unfortunate result of random factors that policing practices result in no white students with law knowledge being arrested. These two cases differ significantly from one another. An adequate description of the causal process leading to the unequal false positive rate in SUNDIAL* cannot ignore that racist police practices cause the measurement bias; hence, in this case membership of a racial category does affect SUNDIAL’s prediction of risk. Accordingly, K-discrimination correctly labels SUNDIAL* as a potential case of morally wrongful discrimination. Conversely, in SUNDIAL** we may plausibly reject that morally wrongful discrimination occurs, since the representation bias with regards to race is merely a random (though perhaps unfair) outcome of an imperfect sampling. Accordingly, K-discrimination correctly rules this case out from the set of potential candidates for discrimination. We believe that this verdict is precisely what we should expect from an adequate theory of discrimination.

A final variety of counterexamples will serve to clarify the conditions of existence of pro tanto wrongful discrimination.^3^ Consider a screening system designed to reject applications by workers with immigrant status but where no workers actually apply. One might want to call the screening system discriminatory. However, K-discrimination as we define it implies that discrimination does not take place in this case, since, by hypothesis, the causal process in question has not produced a single discriminatory outcome. Although this may seem objectionable, the verdict is justified by the observation that, lacking any applicants, the causal process is never triggered. Hence, there is no individual such that, had they been members of a different group, they would not have received the same treatment with the same probability. In other words, we have merely the setup for a discriminatory causal process. By contrast, if we had imagined that a non-immigrant worker had applied and was offered a position, K-discrimination immediately delivers the verdict that the screening system produces discrimination and can be wrong for that reason. This is because had the worker possessed immigrant status instead, they would have not been treated in the same way and with the same probability. Note that what delivers the verdict of K-discrimination is a fact about the causal process when it is actualized, viz., the counterfactual dependence of the actual outcome from immigrant status. Further questions about how our appeal to causal processes meshes with a plausible moral theory of responsibility for discriminatory act practices will be addressed in ‘The Conceptual Limits of Discrimination’ section.

To summarize, this section has shown that counterexamples to the counterfactual fairness approach fail to extend to K-discrimination. The general strategy used in response has been to distinguish genuine cases of pro tanto wrong discrimination from other ways in which something can be deemed as ‘unfair’. As a result, the known counterexamples in the machine learning fairness debate should not be immediately assumed to concern cases of potential discrimination. This is because, as (Binns 2018, p. 3) has put it, ‘“fairness” as used in the fair machine learning community is best understood as a placeholder term for a variety of normative egalitarian considerations'. Conversely, we think that there are distinctive features of discrimination that set it apart from other forms of unfairness (including other forms of group unfairness); K-discrimination plausibly identifies one such feature.

## The Necessity of K-Discrimination, Part II

We are now going to examine two influential definitions of discrimination in the contemporary philosophical literature, by Lippert-Rasmussen (2014) and Hellman (2008, 2018) respectively. Our goal is to show that our thesis is not already implied by those existing accounts. Furthermore, we will argue that, with respect to those cases in which our account diverges from them, there are reasons to prefer ours. This will help establish the claim that, if a philosophical theory of discrimination does not imply the necessity of K-discrimination, it is subject to important objections.

### Lippert-Rasmussen’s Group Discrimination with a Real or Perceived Trait

Lippert-Rasmussen’s (2014, pp. 26–30) account focuses on group discrimination as the primary target. Of this notion, he claims that it is ‘differential treatment plus something else’ (2018, p. 2), where the latter is spelled out in terms of five additional conditions. Altogether, his clauses are meant to provide a sufficient condition for group discrimination that is ‘prima facie morally wrong' (p. 2), meaning not wrong by definition (either pro tanto or all-things-considered). Importantly, the term ‘prima facie’ in Lippert-Rasmussen’s account modifies ‘morally wrong’ and not ‘discrimination’. That is, the notion of prima facie morally wrongful discrimination of which Lippert-Rasmussen (2014) offers an account is something that is ultima facie discrimination in our conceptual framework, even if it may fail to be ultima facie wrong by Lippert-Rasmussen’s lights when the relevant wrong-making property is not instantiated. For example, assuming a harm-based account, an act or practice that is ultima facie discrimination from the conceptual point of view may turn out to be not ultima facie morally wrong in Lippert-Rasmussen’s sense if it turned out that no person was harmed. As we discussed in ‘Clarifying K-Discrimination’ section, we prefer to frame the relevant distinction regarding the notion of discrimination differently. Specifically, our ‘prima facie’ vs ‘ultima facie’ distinction serves to illustrate the move in reflection from the appearance of discrimination to the realization that something is (or is not) an instance of discrimination, quite independently of the verdicts that any moral theory may provide about its wrong-making features. This relative independence from moral theory serves for us the goal of identifying what we believe is an important conceptual thesis and, indeed, constitutes one of the main reasons for thinking that the satisfaction of K-discrimination is conceptually necessary for discrimination.

Although the target notion of Lippert-Rasmussen’s account is different from ours, a convenient way to translate the conditions of his account into our terminology emerges upon reflection. Specifically, it is possible to reformulate his sufficient condition for prima facie morally wrongful discrimination (in Lippert-Rasmussen’s sense) into a necessary condition for ultima facie discrimination that is at least pro tanto wrongful (in our sense). That is to say, we can treat his ‘differential treatment plus something else’ as a condition that any plausible candidate for a case of discrimination that is morally wrong in its distinctive way must satisfy. This is rather trivially the case because the fact that a causal process satisfies K-discrimination is at least defeasible evidence that that process is pro tanto morally wrongful in the distinctive way of discrimination. In effect, the same mutual translatability of the conditions apply to our account: K-discrimination can be understood both as a necessary condition of ultima facie discrimination that is at least pro tanto morally wrongful (in our sense) and as part of a sufficient condition for prima facie morally wrongful discrimination (in Lippert-Rasmussen’s sense). Accordingly, the question whether Lippert-Rasmussen’s conditions imply the necessity of K-discrimination (and, if not, whether this represents a problem for his account) is a meaningful one. In order to assess it, let us take a closer look at the details of his theory.

Lippert-Rasmussen’s definition of group discrimination includes a causal element that explains what makes something an instance of discrimination. The definition is the following:

X discriminates against Y in relation to Z by Φ-ing if, and only if, (i) there is a property, P, such that (X believes that) Y has P and (X believes that) Z does not have P, (ii) X treats Y worse than Z by Φ-ing, (iii) it is because (X believes that) Y has P and (X believes that) Z does not have P that X treats Y worse than Z by Φ-ing, […] (iv′′) P is the property of being member of a certain socially salient group (to which Z does not belong), […] (v) Φ-ing is a relevant type of act etc., and there are many acts etc. of this type, and this fact makes people with P (or some subgroup of these people) worse off relative to others, or Φ-ing is a relevant type of act etc., and many acts etc. of this type would make people with P worse off relative to others, or X’s Φ-ing is motivated by animosity towards individuals with P or by the belief that individuals who have P are inferior or ought not to intermingle with others. (Lippert-Rasmussen 2014, cit., pp. 15–28)

We will call the form of group discrimination defined above LR-discrimination. The causal element of the definition of LR-discrimination is condition (iii), which Lippert-Rasmussen specifies as:

X treats Y worse than Z by Φ-ing because (X believes that) Y has P and (X believes that) Z does not have P if, and only if, (i) the thought that Y, and not Z, has P is part of X’s direct, *motivating reason* for Φ-ing, or (ii) the fact that Y, and not Z, has P *causally explains* X’s Φ-ing and this in turn is causally explained by the fact that people with P are often treated worse than those without P in the sense given by (i) (Lippert-Rasmussen 2014, cit., p. 38, our italics).

Note that, by Lippert-Rasmussen’s definition, treating Y disadvantageously implies that Y’s possession *or* perceived possession of P is the causal antecedent of X’s Φ-ing. It follows immediately that LR-discrimination does not entail K-discrimination: it is not necessary for LR-discrimination that the discriminatee actually belongs to a socially salient group, as K-discrimination requires; it is sufficient that in the causal process leading to a given treatment the person is believed to be a member of such a group, even when this is false, provided that the other conditions are satisfied. Accordingly, the cases of discrimination that satisfy LR-discrimination but not K-discrimination are those in which the belief that someone belongs to a group explains the differential treatment, but the allegedly discriminated person does not actually belong to that group.^4^ Here is an example:

*What’s in the name Andrea*

Hans is a sexist German employer that is looking for a new software developer in his company. He has screened the CVs of candidates, where neither pictures nor information about gender are explicitly provided. Despite possessing the qualifications and skills as other selected candidates, Andrea, an Italian candidate, is excluded by Hans. This is because Hans thinks that Andrea is a woman—in Germany, ‘Andrea’ is indeed a female name. However, Andrea is in fact a man, as ‘Andrea’ is (typically) a male name in Italy.

According to LR-discrimination, Hans’s decision is prima facie wrongful discriminatory against Andrea as this definition regards an act as discriminatory even when the socially salient trait causing the treatment is merely perceived. This means that LR-discrimination treats Andrea’s case as one of ultima facie discrimination that has the potential to be at least pro tanto wrong.

By contrast, K-discrimination denies this. Andrea would be treated by Hans in the same way both in the actual world in which he is male and in the counterfactual world in which he, being female, would have the same observed traits (leading to a rejection). In this case, we argue that K-discrimination correctly denies that the case of Andrea is one of potential discrimination. Although there is overwhelming evidence that Hans’ selection process is discriminatory against women*,* Andrea’s case is not one of discrimination since he is not a woman. In Hans’s society, the name Andrea is usually a reliable signal of being a woman. However, Andrea’s case shows that, as Weinberger (2022) puts it, there are cases in which the link between signal (e.g., a person’s name) and protected attribute (e.g., gender of job applicants) is interrupted. In our view, Andrea is harmed insofar as he was denied the job and arguably wronged insofar as the decision concerning him was taken on arbitrary grounds. Such harm obviously relates to discrimination conceptually. But, although it might seem like discrimination, it is not discrimination ultima facie*.* The peculiar wronging against Andrea, produced by Hans’s discriminatory set-up, results from Hans’s failed attempt to discriminate (and from a casual process that results in discrimination in the vast majority of cases, where individuals receive gendered names that are correctly interpreted as reliable signals of their genders).^5^ We think it should not be put on a par with the wrong received by a woman applicant that Hans rejected because of her gender.

Granted, it may be objected that (just as it happens on Kusner et al.’s (2017) purely formal approach) one could ascribe Andrea to some newly coined demographic group—such as that of ‘individuals who are occasionally perceived to be women based on their names’.^6^ Under this description, the relevant counterfactual for K-discrimination can be built: had Andrea not belonged to such a group, he would have gotten the job. The upshot would be that LR-discrimination either implies K-discrimination, or, at the very least, differs from it at most in non-philosophically interesting ways. However, this objection is based on a misunderstanding of our proposal. Our notion of ‘socially salient group’ is not merely formal. In particular, we deny that 'individuals who are occasionally perceived to be women based on their names' represent a socially salient group in Andrea’s example. Given the definition of socially salient group as something perceived membership of which is important to the structure of one’s social interactions across a wide range of social contexts, it can be plausibly denied that a set of unrelated accidental appearances are sufficient to make a set of individuals part of a socially salient group. Accordingly, it can be plausibly denied that Andrea is subject to discrimination in the morally and politically relevant sense of the term. The plausibility of this verdict lies precisely with its correctly tracking what sorts of facts about belonging to certain groups makes for ultima facie discrimination. Indeed, one virtue of our account that it does not treat all species of misattribution of socially salient traits on a par. It can thus explain why the moral wrong in Andrea’s case is of a different moral species than (among others) the wrong of individuals who are generally vulnerable to being misgendered.^7^

The above arguments may further be objected as follows. Consider a woman applicant with the same qualifications and skills as the other selected candidates, named Andrea (w-Andrea, for ease of exposition). Hans does not select her. But if w-Andrea had been a man, she would not have gotten the job either since, by assumption, Hans excludes candidates with female names. It is an objection to our account of K-discrimination that this clear case of discrimination is not recognized. We reply that this is an apparent objection that can be avoided by a plausible reconstruction of the causal structure in place. Remember that we are not required to identify the closest counterfactual world where w-Andrea is a man but to carefully select the nearby worlds. These plausibly include all and only those worlds in which Andrea is a name given to women and, more generally, in which some common names are reliable indicators for gender. (If this were not the case, the causal process delivering the discriminatory process would not be in place.) In these nearby worlds, an intervention on gender that sets the gender variable to man reduces the probability that the given name takes the value ‘Andrea’ and increases the probability of some other name, that is either gender neutral or a reliable signal of the opposite gender (e.g., Markus). Thus, the counterfactual analysis that matches the causal model goes as follows: *in Germany, had the applicant w-Andrea been a man, she would not have gotten a typical female name and, for that reason, she would have had higher chances of been selected by Hans (who, by assumption, guesses an applicant’s gender based on their name).* This reasoning arguably captures the core intuition behind the claim of discrimination. Viewed from w-Andrea’s perspective, the most immediate source of moral concern is what would have happened during the candidate selection phase had she been a man with similar job-relevant characteristics, not what would have happened specifically to a male counterpart of her that kept her name. The proposed consideration of all nearby worlds thus helps not only vindicate everyday intuitions but also systematize ordinary ways of thinking about discrimination.

In summary, we have provided an example in which LR-discrimination is arguably overinclusive by treating as discriminatory a case in which a person’s membership to a demographic group is not a cause of the differential treatment. K-discrimination yields a different verdict from LR-discrimination because it requires actual, and not merely perceived, membership of a socially salient group. Andrea’s case is one example in which occasional perception of a person as a member of a socially salient group does not suffice to make that person belong to that group. We believe that examination of this type of case lends support to K-discrimination *vis-à-vis* LR-discrimination. In addition to being ethically well grounded, detaching ultima facie discrimination from what an agent perceives or believes makes our framework immediately extendable to cases (e.g. algorithmic predictions) in which the discriminating agent’s reasoning is opaque or where mental states are not involved in the process leading to a decision.

### Hellman’s Non-Moralized Discrimination with a Real Trait

Hellman (2018, p. 98) provides a non-moralized definition of discrimination, which we call H1-discrimination. It is disjunctive, since in Hellman’s theory, discrimination is either direct or indirect (i.e., it is a form of disparate impact). It goes as follows:

An act or policy (directly) discriminates if the actor or the policy treats person A differently from B on the basis of A having or lacking some trait X. Alternatively, a policy (indirectly) discriminates if the policy has a disparate impact on persons with trait X as compared to persons with trait Y. (Hellman 2018, p. 98)

In what follows, we assume that Hellman’s non-moralized definition of discrimination is intended as a condition of ultima facie discrimination in our sense, i.e., something that has the potential to be pro-tanto morally wrongful in that discriminatory*.* H-discrimination becomes ultima facie discrimination (but still possibly only pro tanto morally wrongful) when two additional conditions occur. First, the act expresses that the discriminatee is less worthy of concern than other people according to a specific social or conventional meaning (2008, pp. 29–30, 33, 35). Second, the discriminator is in a position of power or has a superior status than the discriminatee. A demeaning act is one in which the two above-mentioned conditions occur. We refer to this form of discrimination as H2-discrimination.^8^ H1-discrimination plus the wrong-making features of discrimination is equivalent to H2-discrimination, and, hence, purports to be ultima facie discrimination that is at least pro-tanto morally wrong.

Our argument against Hellman’s account will proceed as follows. First, we will identify a decision that is neither an instance of H1-discrimination, as it is random, nor the result of a causal process, and so cannot be labeled as H2-discriminatory either. Then we will argue that, if Hellman’s account correctly classifies the case as non-discriminatory, it can only do so by appealing to either of two things: to an implicit causal conceptual criterion, on the one hand, or to the wrong-making properties explicitly invoked in her conditions, on the other. However, if she chooses the latter option, she ends up in trouble. Hence, in order to explain why a discriminatory practice has the wrong-making features it does, one must invoke K-discrimination—or so we shall argue. In this way, we justify the claim that H2-discrimination is not an adequate account of ultima facie discrimination that is at least pro tanto wrong—or not so until K-discrimination is appealed to.^9^ The supporting case is the following:

*The lottery*

Consider a non-biased lottery that for two years has been used to select top managers in large corporations. The selection is between the top two candidates, who have been ranked as equally meritorious. Let us assume that, every year, of the two top candidates, one is a man and the other a woman. However, observing the statistics of the lottery we find that 60% of the winners are men.

We contend that ‘the lottery’ is, intuitively, not an instance of ultima facie discrimination that is at least pro tanto wrongful discrimination. The problem is the verdict that Hellman’s account would provide of this case. There are at least two possible responses to consider, depending on the interpretation one gives to Hellman’s conditions. Let us consider them in detail.

On the first response, the lottery does not have a disparate impact on women, because the concept of ‘disparate impact’ should never consider only the observed actual distribution of outcomes, but also whether the mechanism in place is likely to distribute outcomes unequally in the long term. When an outcome disparity produced by mechanism M is clearly due to chance, M should not for that reason be considered discrimination. Accordingly, on this reading, Hellman would be arguing that ‘the lottery’ is not an instance of discrimination in the ultima facie sense: it is not even a candidate to be morally wrong qua discrimination because, irrespective of moral wrongness, it is not even an instance of H1-discrimination; a fortiori, it cannot be an instance of H2-discrimination, either.

On this interpretation of the lottery’s case, the claim that Hellman should defend is that the disparate distribution of outcomes is due to chance, since we have robust a priori reasons to believe that the mechanism producing the result is a random one. This is, in our view, the only reason, if any, why one should agree that the process in question should not be considered discriminatory ultima facie. That, basically, amounts to denying the possibility that the “disparate impact” in the sense that matters for the definition of H1 discrimination can be due to chance. In other words, disparate impact must be considered as something that can be explained in counterfactual terms by the influence of group membership on the outcome. So, the reason why the lottery should not be considered an instance of ultima facie discrimination is the fact that a counterfactual condition is not satisfied. This condition, as we shall further argue in the next section, is best understood in terms that imply K-discrimination.

On an alternative response to the lottery objection, Hellman might reply that ‘the lottery’ produces a disparate impact on women as compared to men and is for that reason an instance of (indirect) H1-discrimination. However, ‘the lottery’ does not pass the further test that is necessary to qualify as H2-discrimination. Indeed, the lottery does not convey a demeaning message towards the discriminated group (women, in this case) coming from somebody in a position of power. Presumably, this could be used to explain why we do not intuitively take the lottery to be a genuine case of discrimination.

However, just as in the previous interpretation of Hellman’s conditions, we contend that on this reading Hellman’s account avoids classifying the lottery case as one of genuine discrimination only insofar as it implicitly appeals to a notion of counterfactual dependence on group membership. To see this, suppose that, upon further examination, the lottery is found to be rigged in favor of men. Surely, this would convey a demeaning message toward women and the case would be immediately recognized as one of discrimination. But notice that by offering this explanation of what would make the process demeaning, we are comparing the original lottery case with one in which the mechanism was set up in such a way that it would allow gender to have a causal influence on the outcome. This is, after all, what ‘rigged in favor of men’ amounts to. Consequently, the wrong-making property (being demeaning) which could make ‘the lottery’ an instance of H2-discrimination is instantiated necessarily by a process with the property of being rigged in favor of one group. And this necessarily implies the possibility of a causal influence of the group on the outcome. In other words, although Hellman’s account of H2-discrimination avoids classifying ‘the lottery’ as prima facie morally wrong, it can only do so by implicitly assuming that a relevant counterfactual condition is not met.

To sum up, Hellman’s definition of discrimination can indeed classify cases of discrimination in a way that aligns with what it would achieve if it invoked K-discrimination explicitly. However, her theory is incomplete: it cannot explain what differentiates an instance of wrongful discrimination from one that is not, without implicitly invoking a counterfactual condition. In fact, both when the lottery is interpreted as a non-ultima facie instance of discrimination and as an ultima facie instance of discrimination without the relevant wrong-making features, the explanation provided by Hellman must necessarily point to the causal role of group membership and, hence, to K-discrimination.

## The Conceptual Limits of Discrimination

Until this point, we have argued that a connection of counterfactual dependence between the individual or group that is being discriminated against and the outcome of a discriminatory causal process is necessary for ultima facie discrimination that is at least pro tanto morally wrongful. Yet, it may be objected, there may be causal relations between one’s perceived membership of a group and one’s treatment that are relevant to assessing discriminatory practices but are not capturable in counterfactual terms. This is, indeed, one of the prominent worries raised against the original counterfactual fairness approach (see, e.g., Hu and Kohler-Hausmann 2020; Kohler-Hausmann 2019; Sen and Wasow 2016). To understand the criticism, we must go back to the fact that, on our view, an individual is subject to discrimination only if, by intervening solely on the group membership variable and all other variables causally downstream from it, a causal process does not yield the same outcome with the same probability. However, as Woodward (2003) explains:

Something cannot be a causal predicate in the counterfactual framework if we cannot coherently describe what it would be like for the relevant intervention to occur at all or for which there is no conceivable basis for assessing claims about what would happen under such interventions because we have no basis for disentangling, even conceptually, the effects of changing the cause variable alone from the effects of other sorts of changes that accompany changes in the cause variable. (Woodward 2003, p. 132)

This fact poses a problem for K-discrimination if it is true that perceived membership to gender and race are, in some sense, ‘structural’ causes: more precisely, if it is impossible to disentangle, even conceptually, the effects of changing a protected attribute such as race or gender from the effects of other sorts of changes—including those that, according to the relations of counterfactual dependence that are assumed to hold in a given situation, predict the outcome of interest. This is precisely the view that Kohler-Hausmann has defended in a recent attack on counterfactual fairness:

Inhabiting a particular racial category not only shapes the opportunities, advantages, and resources that will be available in a person’s life course, it means living with a particular cultural meaning attached to one’s body. [...] It is impossible (that is, illogical, nonsensical, improbable, meaningless) to ask of the person: Be the exact same unit *except for race*, but do not change anything else about yourself because I want to see the effect of race and race alone on an outcome. (Kohler-Hausmann 2019, pp. 1204–1205)

The alternative framework that Hu and Kohler-Hausmann (2020) propose involves the use of ‘constitutive’ diagrams, which investigate discrimination on the basis of a type of counterfactual that is different from the causal one adopted in the counterfactual fairness approach. Constitutive diagrams examine the constitutive or definitional relations between a protected attribute and its features.

On our view, however, Kohler-Hausmann’s constitutive objection does not offer a sufficient reason for abandoning the K-discrimination approach. Our response is twofold. On the one hand, we believe that it is often meaningful, pace defenders of the constitutive objection, to disentangle how certain aspects of gender or race causally affect others. Granted, when we ask how (say) being a woman affects one’s chances of managerial success in a society, it would be wrong to simply identify a candidate woman manager and ask how well a man with the same traits would have fared. For, clearly, the typical characteristics that make one successful as a manager as a woman may not be the same as those conducive to managerial success in men, and vice versa. This is not, however, the kind of comparison that K-discrimination urges us to make. For not only the intervention variable (i.e., the group membership feature) but all variables causally downstream from it must be adjusted in the counterfactual assessment. For instance, empirical evidence may indicate that assertiveness constitutes a contributing factor for managerial success in men, but less so in women. In such a case, it would be necessary to consider how a woman would have fared in terms of managerial success had she been a man, when differences in assertiveness are appropriately compensated. It is precisely the job of the causal modeler to identify, to the best of their possibilities, the relevant variables to control in order to test how a person’s socially salient group affects the distribution of other goods.^10^

On the other hand, we concede that there are cases in which the question regarding the dependence of an outcome from group membership is so holistic that it is not representable in terms of a ‘nearby’ counterfactual world. Specifically, one might want to point to forms of unjust treatment of women that can only be identified by comparing the treatment that a woman receives in the actual world with that which they would have received in a world in which gender roles were structured in a completely different way (or where gender did not exist altogether). When considering such cases, our approach may appear to deliver an objectionable narrowing down of the scope of discrimination, losing sight of the broader picture of all the harm or wrongs produced by the existing socially salient categories. Here, however, we bite the bullet: despite appearances, the ‘distant’ counterfactual worlds that the objection highlights are irrelevant to the question of what constitutes discrimination. Indeed, the restriction of the counterfactual comparison to the ‘nearby’ worlds in which the actual world’s causal structure remains intact—in which the same cultural meanings are attached to perceived group memberships—is a feature and not a bug of our account. In what follows, we will outline three arguments for this claim.

First, if distant counterfactual worlds are admitted, there is a problem about where our consideration of them should stop. For example, it would be very hard to say what outcome a certain procedure would have produced in a matriarchal society among hunter-gatherers. But it seems that the consideration of such a counterfactual world, and a great number of others, cannot be ruled out a priori if we set out to investigate the effects of gender or race categorizations on people’s treatment with such a broad view of the relevant alternatives. Imagining distant possibilities would therefore make it harder to identify the most immediate and salient causal effects of group membership, making rightness and wrongness extremely difficult to ascertain, even conceptually. A more sensible view is therefore to restrict our attention to understanding how, given the causal structure of the social world we live in, a specific causal process may realize an outcome of discrimination.

Second, and relatedly, discrimination is an element in the current moral vocabulary possessing strong connections with the idea of moral and legal responsibility. If distant counterfactual worlds were to be admitted in the relevant counterfactual comparison, these connections would start blurring. After all, removing discrimination would appear to depend on the possibility of altering the social context in ways that are so broad and holistic as to be outside the sphere of what is imaginable as individual responsibility. The comparison with far-away possible worlds would also be an impediment to identifying fine-grained individually discriminatory outcomes, such as actions or decisions, and specific discriminatory processes as distinct from a more generalized influence of socially salient categories and hierarchies on societal outcomes. The advantage of K-discrimination *vis-à-vis* the constitutive alternative thus consists in ensuring that there is something, i.e., an actual causal process delivering some outcomes*,* to which individual responsibilities (of both the moral and the legal kind) can typically be attached, at least when it is plausible that they may exist.

Third, and finally, there is an advantage to understanding discrimination as a specific, rather than a generic, type of unfairness. By means of K-discrimination, we can identify narrow targets for policies to modify or ameliorate. Admittedly, this approach may appear less plausible to those who view the fight against discrimination as a radical transformation of social relations, rather than a form of piecemeal social engineering. We understand their concern, but we reply that other forms of group injustice can be defined without necessarily borrowing the vocabulary of discrimination. We may still deem the actual world as ‘unjust’ in some sense when compared to a world in which basic social meanings and facts were altogether different from ours. We do not claim that ethics primarily consists of an account of the narrow, legalistic rights and wrongs and we are thus open to the view that the concept of a good or well-ordered society should take logical priority in our thinking about justice. We only contend that discrimination, as it is understood in current moral practices, is best thought of as an element of the part of morality that deals with what is right and wrong in the strict sense. The benefits of stretching the notion of discrimination beyond these limits are not worth the costs.

In summary, we have outlined three arguments for why the overall way in which gender or race influence social relations should be assumed to be constant across all possible worlds relevant to assessing potential cases of discrimination. In those cases in which the effect of a socially salient group on decisions is so holistic that it cannot be represented by nearby counterfactual worlds, e.g., when it can only be explained through a comparison with distant possibilities, the potential moral wrong in question ceases to be one of discrimination. Importantly, in making our argument we do not conflate morally wrongful acts of discrimination with what an individual may be deemed responsible for. We may suppose that there are cases of discrimination in which it is hard, or even impossible to ascribe responsibilities to individuals—for instance, when there is insufficient evidence for a discriminatory process, or when all individuals involved in bringing about a certain outcome cannot help but discriminate.^11^ Our claim is solely that as the result of what we want ‘discrimination’ to do in our moral vocabulary it is sensible to think of it as conceptually limited to the idea of differential treatment relative to a particular portion of the logical space of alternatives. We conclude, then, that K-discrimination is a genuine conceptual requirement on discrimination.

## Conclusion

In this contribution, we have contended that discrimination implies that a counterfactual condition is met. Roughly, this means that for an individual to have a claim to be discriminated against by a given procedure or practice, it must be the case that, had that individual belonged to a different socially salient group (e.g., gender, race), they would not have received the same treatment with the same probability. We defined ‘K-discrimination’ as the result of any causal process that does not bring about the same treatment with the same probability in the actual world and in a nearby counterfactual world where the individual belongs to another socially salient group. As a way of clarifying and defending our thesis, we have introduced a novel distinction between prima facie discrimination (i.e., what appears to be discrimination) and ultima facie discrimination (i.e., what is truly discrimination) and further distinguished between pro tanto wrongful discrimination and all-things-considered wrongful discrimination. On the basis of these distinctions, we have contended that K-discrimination is a necessary condition for ultima facie discrimination that is at least pro tanto morally wrong.

We have derived the counterfactual condition of K-discrimination from Kusner et al.’s (2017) proposal of counterfactual fairness. While such a proposal was criticized in the debate of algorithmic fairness (Castro 2022; Castro et al. 2022; Chiappa 2019), its relevance for discrimination has been underestimated. In this contribution, we have argued that the notion of K-discrimination is philosophically relevant because, before discussing the wrong-making properties of an alleged discriminatory acts (as it is common in the current philosophical debates on discrimination), one should clarify what discrimination is. Before any theory-laden element is attached to discrimination, K-discrimination identifies a conceptual core of the concept of discrimination, namely, a necessary condition for an act or practice to be discriminatory. Therefore, K-discrimination enables one to identify acts or practices of discrimination in the real world and to substantiate claims about alleged discriminatory acts or practices. Also, while counterfactual fairness is subject to counterexamples in which the conditions for fairness are (un)met but the intuitive verdict diverges (Castro et al. 2022), we have argued that K-discrimination is not subject to such counterexamples as the latter concern a core element of discrimination and not of fairness. As a matter of fact, fairness is a broad term including various egalitarian considerations, of which discrimination is a specific type.

We have demonstrated the necessity of the counterfactual condition for discrimination by showing that two of the most prominent accounts of morally wrong discrimination—Lippert-Rasmussen’s and Hellman’s—face significant objections if they do not include the counterfactual condition. More precisely, Lippert-Rasmussen’s account is overinclusive since it considers as cases of discrimination those that are wrong and connected to discrimination but not discriminatory. By contrast, K-discrimination enables one to distinguish instances of wrongful discrimination from acts or processes that are wrong and connected to discrimination but not discriminatory. This is due to the main philosophically relevant difference between the two accounts: K-discrimination is based on actual membership of socially salient groups, whereas Lippert-Rasmussen’s account on perceived *or* actual membership of them. Hellman’s account is immune to the objection of over-inclusivity only so long as it implicitly appeals to a counterfactual account of what makes something an instance of discrimination, which is the influence of socially salient groups on the treatment of an individual.

K-discrimination is relevant to the philosophical debate on discrimination insofar as it helps define the conceptual limits for claims about discriminatory practices in society. At the same time, K-discrimination is relevant to the debate over measures of algorithmic fairness as it focuses on a specific form of unfairness—i.e., discrimination—which is conceived as the result of a causal process which need not be attributable directly to an agent’s perceptions, beliefs or reasoning. As a result, it can countenance those rapidly growing forms of discrimination caused by opaque prediction algorithms.

## Footnotes

1 We are not implying that absent K-discrimination there can be no causal influence of group membership.

2 Chiappa’s (2019) path-dependent counterfactual fairness approach can address Castro’s problem of the Spanish test by treating the path from being a native speaker to passing the test as a permissible path of causal influence.

3 Many thanks to an anonymous reviewer for raising this potential objection to our thesis.

4 Note that this is possible even though we have defined a ‘socially salient group’ as one such that ‘*perceived* membership to it…’ (following Lippert-Rasmussen (2014)). The case of Andrea discussed below illustrates that the conditions for perceived membership to a given demographic group go well beyond being accidentally mistaken for a member of that group in a given occasion (or even a series of occasions).

5 The existence of peculiar species of moral wrongness that are conceptually related to discrimination but are not the moral wrongs of discrimination cannot be reasonably considered evidence against K-discrimination being too narrow as a necessary condition of discrimination. For generally, the fact that the nature of X can only be explained by referring to the nature of Y does not imply that every X is an instance of Y.

6 We thank two anonymous referees of the paper for raising this objection.

7 It is important to stress that not all groups defined by social appearance fail to be, for that reason, socially salient. The group of people that are only rarely misidentified as women is not on a par with the group of people who are regularly misgendered, as may be the case with transgender individuals. There is an important difference in terms of social salience, as being part of a group that is regularly or typically misgendered is a condition that shapes social interactions across a wide range of social contexts.

8 A charitable reading of Hellman implies that this form of discrimination is only pro tanto morally wrong. For it is always possible that a given demeaning act is ultimately permissible or even required because of the circumstances. For example, an H2-discriminatory treatment may be demeaning and yet all-things-considered morally permissible if strictly necessary to save millions of lives.

9 This falls short of showing that Hellman’s definition should presuppose the counterfactual condition on discrimination to avoid being overinclusive. However, the gap between the argument for including a causal criterion (that we provide here) and the argument for K-discrimination will be closed in the next section.

10 When a model does not recognize any path of influence from a socially salient group to the outcome, it should not be immediately concluded that discrimination does not exist. For it is also possible that the model is incomplete. K-discrimination is an objective, not an epistemic condition: it is defined relative to an ideal model, which is the model that captures all the relevant causal influences (that can be modeled counterfactually according to the above-mentioned restrictions about possible worlds) in the real world.

11 We thank an anonymous reviewer for signaling this case.
